# Beta 1-integrin ligation and TLR ligation enhance GM-CSF–induced ALDH1A2 expression in dendritic cells, but differentially regulate their anti-inflammatory properties

**DOI:** 10.1038/srep37914

**Published:** 2016-11-29

**Authors:** Aya Yokota-Nakatsuma, Yoshiharu Ohoka, Hajime Takeuchi, Si-Young Song, Makoto Iwata

**Affiliations:** 1 Laboratory of Immunology, Kagawa School of Pharmaceutical Sciences, Tokushima Bunri University, Shido, Sanuki-shi, Kagawa, Japan; 2 Japan Science and Technology Agency, CREST, Chiyoda-ku, Tokyo, Japan; 3 Institute of Neuroscience, Tokushima Bunri University, Shido, Sanuki-shi, Kagawa, Japan

## Abstract

Retinoic acid (RA)–producing CD103^+^ mature dendritic cells (DCs) in mesenteric lymph nodes (MLNs) play crucial roles in gut immunity. GM-CSF and RA contribute to the expression of the RA-producing enzyme ALDH1A2. However, additional signals appeared to be required for inducing ALDH1A2^high^ mature DCs from immature DCs. We found here that TLR ligands (Ls) and immobilized E-cadherin could provide such signals in FLT3-L–generated bone marrow (BM)–derived DCs after treatment with GM-CSF and the RA receptor agonist Am80. The TLR-L-treated DCs produced proinflammatory cytokines unlike normal ALDH1A2^high^ MLN-DCs, whereas the E-cadherin-treated DCs did not. Immobilized VCAM-1 and semaphorin 7 A exerted effects similar to those of E-cadherin. Soluble anti-integrin β1 antibodies or inhibitors of integrin signaling molecules suppressed the effects of these immobilized proteins, whereas immobilized anti-integrin β1 antibodies enhanced the GM-CSF/Am80-induced ALDH1A2 expression without inducing proinflammatory cytokines. Sequential stimulation of splenic pre-DCs with GM-CSF/Am80 and immobilized E-cadherin or anti-integrin β1 antibody also induced differentiation to mature DCs with high ALDH activity. The E-cadherin-treated BM-DCs induced gut-tropic Foxp3^+^ T cells and alleviated DSS–induced colitis, whereas the TLR-L-treated DCs aggravated DSS–induced colitis. The results suggest that integrin β1-mediated signals contribute to the differentiation and maturation of RA-producing anti-inflammatory DCs.

Retinoic acid (RA), an active vitamin A metabolite, plays important roles in gut immunity. Dendritic cells (DCs) in the gut-related lymphoid organs, mesenteric lymph nodes (MLNs) and Payer’s patches (PPs) produce RA and imprint gut-homing specificity on T and B cells upon antigenic stimulation^1^,^2^. RA also regulates the functional differentiation of T cells. RA suppresses the differentiation of Th1 and Th17 cells via RA receptors (RARs)^3^,^4^,^5^,^6^,^7^, although it has recently been reported that RA signaling is essential for Th1 cell lineage stability and preventing the conversion of Th1 to Th17^8^. On the other hand, RA enhances the TGF-β-dependent differentiation of naïve CD4^+^ T cells into Foxp3^+^ inducible regulatory T cells (iTregs)^4^,^5^,^6^,^7^,^9^,^10^,^11^,^12^. Thus, RA has been implicated in the induction of oral tolerance. Indeed, vitamin A deficiency results in the failure to tolerate oral antigens^13^. RA appears to regulate the nature of MLN-DCs as well as that of T cells primed in MLNs^13^,^14^,^15^,^16^.

Retinal dehydrogenases [e.g., aldehyde dehydrogenase 1A (ALDH1A)] encoded by the *Aldh1a* gene family are key enzymes that produce RA, and they are expressed in limited cell types. RA-producing DCs in MLNs, PPs, and the small intestinal lamina propria (LP) express the ALDH1A2 isoform at different levels^1^,^14^,^17^. These ALDH1A2^+^ DCs are included in the CD103^+^ DC subset^9^,^14^. As most CD103^+^ MLN-DCs migrate from the LP^18^, it is probable that ALDH1A2^+^ MLN-DCs mostly originate from the LP. Gut-homing DC precursors, such as lineage^−^CD11c^int^B220^+^α4β7^+^CCR9^−^ cells, develop in the bone marrow (BM) and migrate into the intestinal LP^19^. The microenvironment of the LP appears to induce ALDH1A2 expression in immature DCs. We previously found that GM-CSF induces ALDH1A2 expression in FMS-like tyrosine kinase 3 (FLT3) ligand (L)–generated immature BM-DCs^14^,^20^. RAR-mediated signaling is required for GM-CSF–induced ALDH1A2 expression, although RA by itself only weakly induces *Aldh1a2* expression^14^. In the LP, RA may be produced by ALDH1A1^+^ epithelial cells, ALDH1A2^+^ DCs, and subpopulations of macrophages and stromal cells^1^,^21^,^22^, whereas GM-CSF may be produced by a variety of cell types, including macrophages and stromal cells, in an RA-dependent manner^14^,^21^.

ALDH1A2^high^ MLN-DCs are mature DCs^14^. GM-CSF and RA are not sufficient to generate mature DCs with high ALDH1A2 activity from BM-DCs *in vitro*^14^,^23^. We previously illustrated that the simultaneous stimulation of FLT3-L–generated BM-DCs with GM-CSF and Toll-like receptor (TLR)-L induced their maturation and high levels of ALDH1A2 expression^14^. However, we found here that they produced proinflammatory cytokines, unlike steady-state CD103^+^ALDH1A2^+^ MLN-DCs in SPF mice. Thus, we searched for factors that are present in the intestinal tissue environment and enhance ALDH1A2 expression in BM-DCs without inducing proinflammatory cytokine production. We found that stimulation of FLT3-L–generated BM-DCs with GM-CSF/RA and subsequent stimulation with immobilized E-cadherin efficiently generate RA–producing anti-inflammatory DCs that can induce iTreg differentiation, and that integrin β1-mediated signals contribute to the E-cadherin effect.

## Methods

### Mice

C57BL/6 mice and TCR-transgenic OT-II/Rag2^−/−^ mice were obtained from CLEA Japan (Tokyo, Japan) and Taconic (Hudson, NY), respectively. GM-CSF-deficient (GM-CSF^−/−^) mice were kindly provided by Drs. Jeffrey A. Whitsett and Bruce C. Trapnell (Children’s Hospital Medical Center, Cincinnati, OH) with kind permission of Dr. Glenn Dranoff (Dana Farber Cancer Institute, Boston MA). All animal experiments were performed according to protocols approved by the Animal Care and Use Committee of Tokushima Bunri University (Tokushima–Kagawa, Japan; permit numbers: KP11-41-003a, KP12-41-001a, KP12-41-002, KP13-41-001, KP13-41-002, KP14-41-001, KP14-41-002, KP15-41-001, KP15-41-002, and KP-16-41-001).

### Antibodies

The anti-integrin β1 monoclonal antibody (mAb), KMI6, was purchased from OriGene Technologies (Rockville, MD). Blocking mAbs to integrin α1 (clone HMα1), α4 (R1-2), α5 (5H10-27), β1 (HMβ1-1), and β7 (FIB27) were from BioLegend (San Diego, CA); αE/CD103 (M290) and β1 (9EG7) were from BD Biosciences (San Jose, CA). The following mAbs were used for flow cytometry and cell sorting: CD11c (N418), CD40 (1C10), CD86 (GL1), CD103 (2E7), integrin α1 (HMα1), α2 (HMα2), α4 (R1-2), α5 (5H10-27), α6 (GoH3), αV (RMV-7), β1 (HMβ1-1), β7 (FIB504), α4β7 (DATK32), IFN-γ (XMG1.2), and IL-17A (TC11-18H10.1) were from BioLegend; E-cadherin (114420) and CCR9 (242503) were from R&D Systems; CCR7 (4B12) and Foxp3 (FJK-16s) were from eBioscience (San Diego, CA); CD16/CD32 (2.4G2) and I-A^b^ (AF6-120.1) were from BD Biosciences.

### Cell isolation and culture

DCs were isolated from the small intestinal LP, PPs, MLNs, and spleens (SPLs) using MACS (Miltenyi Biotec, Bergisch Gladbach, Germany) as previously described^14^. BM-DCs were generated by culturing BM cells for 7 days in the presence of 20% culture supernatant of a mouse FLT3-L-transfected CHO cell line. For isolation of pre-DCs, CD11c^+^ cells were isolated with MACS from SPLs of C57BL/6 mice that had received an s.c. injection of FLT3-L-transfected B16-F10 (B16-FLT3L) cells, and CD11c^+^I-A^b−^CD103^−^ pre-DCs were collected by sorting with a FACSAria (BD Biosciences). BM-DCs or pre-DCs were cultured for 2 days in the presence of GM-CSF (10 ng/ml; Peprotech, Rocky Hill, NJ) and all-*trans*-RA (10 nM) (Sigma-Aldrich, St Louis, MO) or Am80 (10 nM) kindly provided by Dr. Hiroyuki Kagechika (Tokyo Medical and Dental University, Tokyo, Japan) with or without TGF-β (0.5, 5 ng/ml; R&D Systems, Minneapolis, MN) and subsequently stimulated for 1 day in new plates with Pam3CSK4 (1 μg/ml), CpG oligonucleotide (ODN) 1826 (0.1 μM), curdlan (100 μg/ml) (all from InvivoGen, San Diego, CA), LPS (1 μg/ml; Sigma-Aldrich), or in plates coated with E-cadherin/Fc chimera protein, mucosal addressin cell adhesion molecule-1 (MAdCAM-1)/Fc, vascular cell adhesion molecule-1 (VCAM-1)/Fc, semaphorin 7 A/Fc (5 μg/ml; all from R&D Systems), or the anti-integrin β1 mAb, KMI6 (10 μg/ml). In selected experiments, GM-CSF/Am80-treated BM-DCs were pretreated for 15 min with blocking mAbs to integrins (50 μg/ml) or inhibitors of focal adhesion kinase (FAK) (PF 573228, 5 μM; Adooq Bioscience, Irvine, CA), Akt (triciribine, 5 μM; Wako Pure Chemical Industries, Osaka, Japan), PI3K (LY294002, 5 μM), β-catenin (ICG-001, 5 μM) (both from Cayman Chemical, Ann Arbor, MI), mammalian target of rapamycin (mTOR) (rapamycin, 0.1 μM; Cell Signaling Technology), MEK (PD98059, 50 μM), or NF-κB (1 μM) (both from Calbiochem, San Diego, CA).

Naïve CD4^+^CD62L^+^ T cells were obtained from lymph nodes and SPLs of OT-II/Rag2^−/−^ mice by negative selection using EasySep Mouse CD4^+^ T Cell Enrichment Kits (Stemcell Technologies, Vancouver, Canada) and subsequently by positive selection with CD62L Microbeads (Miltenyi Biotec). DCs were pretreated for 2 h with OVA peptide P323–339 (1 μM), washed, and then cocultured for 5 days with naïve CD4^+^CD62L^+^ T cells (2 × 10^4^) at a ratio of 1:1. For induction of regulatory T cells, cultures were supplemented with TGF-β (2 ng/ml).

### Real-time PCR

Total RNA was isolated from cells using RNeasy Mini Kit, and cDNA was generated using QuantiTect Reverse Transcription Kit (both from Qiagen, Valencia, CA). cDNA was used as a template for real-time PCR in triplicates with Power SYBR Green PCR Master Mix (Applied Biosystems Japan, Tokyo, Japan) and gene-specific primers for *Aldh1a2* (forward: 5′-TGGGTGAGTTTGGCTTACGG-3′ and reverse: 5′-AGAAACGTGGCAGTCTTGGC-3′) or *Klrg1* (forward: 5′-TGCAGACAAAGGCTCACATC-3′ and reverse: 5′-ACCTCCAGCCATCAATGTTC-3′). Analysis was performed on an Applied Biosystems 7900 Real-time PCR system. The expression of *Aldh1a2* and *Klrg1* was normalized with *Rplp0* (forward: 5′-GGTGCCACACTCCATCATCA-3′ and reverse: 5′-CGCAAATGCAGATGGATCAG-3′), and relative expression level was quantified with the 2^−ΔΔ*Ct*^ value unless otherwise indicated.

### Flow cytometry

ALDH activity in individual cells was estimated using ALDEFLUOR staining kits (Stemcell Technologies) as previously described^14^. Cells were stained with indicated mAbs conjugated to various fluorochromes in the presence of anti-CD16/CD32 mAb. Intracellular staining of Foxp3 was performed using mouse/rat Foxp3 staining sets (eBioscience). For intracellular cytokine staining, CD4^+^ T cells obtained from cultures were restimulated for 5 h with PMA (50 ng/ml) and ionomycin (750 ng/ml) (both from Calbiochem). Monensin (3 μM; Sigma-Aldrich) was added to the cultures for the last 2 h. After surface staining, the cells were fixed with Fixation Buffer (BioLegend), and intracellular cytokine staining was performed according to the manufacturer’s protocol. Analysis was performed on a FACSAria or FACSCalibur with CellQuest Pro software (BD Biosciences).

### Elisa

Cytokine concentrations in the culture supernatants were assessed with ELISA kits for IFN-γ, IL-6, IL-12p40 (BD Biosciences), IL-17A, IL-23p19, and TNF-α (BioLegend).

### Dextran sulfate sodium (DSS)–induced acute colitis

Colitis was induced by administration of DSS as described previously^24^ with slight modifications. Briefly, male C57BL/6 mice aged 8–9 wk were administered 2% DSS (m.w. 36,000–50,000; MP Biochemicals, Solon, OH) in their drinking water ad libitum for 4 days followed by feeding with regular drinking water. The mice were injected intraperitoneally with BM-DCs (1 × 10^6^ in 0.1 ml of PBS/mouse) or PBS alone on days 0 and 2, and their body weights were recorded daily. The mice were sacrificed on day 10, and their colon lengths were assessed.

### Statistical analysis

Statistical comparisons were performed using the one-way ANOVA with Tukey–Kramer multiple comparisons test and the two-tailed unpaired Student’s *t* test. Values less than 0.05 were considered statistically significant.

## Results

### Two-day stimulation with the combination of GM-CSF and RAR ligands induces both CD103 and ALDH1A2 expression in FLT3-L–generated BM-DCs, and subsequent stimulation with E-cadherin significantly enhances the ALDH1A2 expression without inducing proinflammatory cytokines

**Figure 1:**
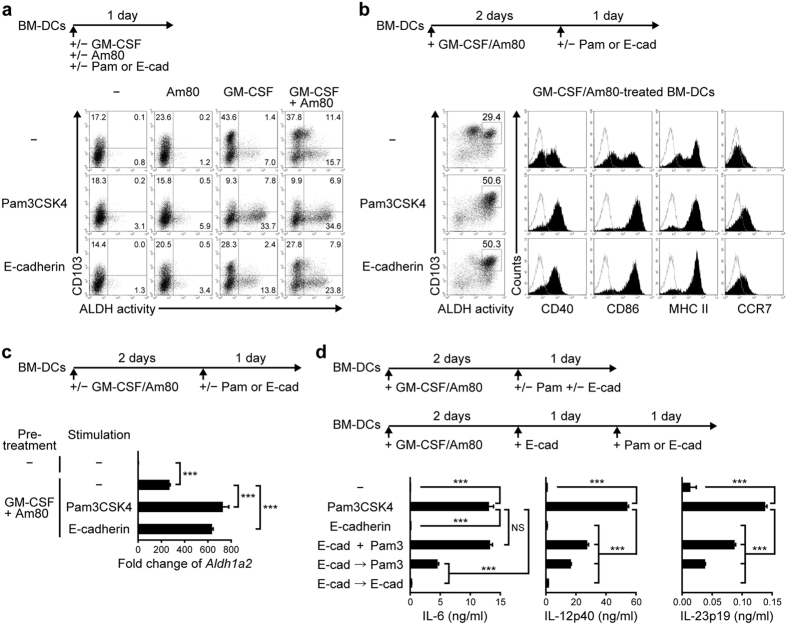
Delayed stimulation with TLR-L or E-cadherin of GM-CSF/Am80-treated BM-DCs enhances their ALDH1A2 expression without reducing CD103 expression. (**a**) Representative flow cytometric profiles of the ALDH activity and surface CD103 expression of BM-DCs cultured for 1 day with Pam3CSK4 or immobilized E-cadherin/Fc in the presence or absence of GM-CSF and Am80. (**b**–**c**) BM-DCs were cultured for 2 days with GM-CSF and Am80 and subsequently stimulated for 1 day with Pam3CSK4 or immobilized E-cadherin/Fc. (**b**) Representative flow cytometric profiles of ALDH activity and expression of the indicated surface molecules. Solid lines represent isotype controls. (**c**) *Aldh1a2* expression was assessed by real-time PCR. Relative expression levels are presented as the mean + SD of triplicate samples relative to that of the cells cultured in medium alone. (**d**) Effects of simultaneous or sequential stimulation of GM-CSF/Am80-treated BM-DCs with E-cadherin/Fc and Pam3CSK4 on the production of IL-6, IL-12p40, and IL-23p19. GM-CSF/Am80-treated BM-DCs were stimulated for 1 day with Pam3CSK4 and immobilized E-cadherin/Fc or each one alone. Aliquots of the E-cadherin/Fc-treated BM-DCs were further stimulated for 1 day with Pam3CSK4 (E-cad → Pam3) or E-cadherin/Fc (E-cad → E-cad). Cytokine concentrations in the culture supernatants were assessed by ELISA. Results are presented as the mean + SD of triplicate samples. Statistical significance was determined by the one-way ANOVA with Tukey–Kramer multiple comparisons test. ***p < 0.001. Data are representative of three independent experiments.

In FLT3-L–generated BM-DCs, GM-CSF and the RAR agonist Am80 synergistically enhanced *Aldh1a2* expression and ALDH activity in BM-DCs after 1 day of culture (Fig. 1a). Simultaneous stimulation of BM-DCs with GM-CSF and the TLR2/1-L Pam3CSK4 in the presence or absence of Am80 induced high levels of ALDH activity, as we previously reported^14^, but reduced CD103 expression (Fig. 1a). On the contrary, stimulation of BM-DCs with only the combination of GM-CSF and Am80 for 2 days followed by replating (transferring the cells to new culture plates) and 1-day culture enhanced ALDH activity (Fig. 1b). Replating by itself moderately enhanced the effect of GM-CSF/Am80 on ALDH activity and the expression of maturation markers (data not shown). The addition of Pam3CSK4 to the replated culture markedly enhanced ALDH activity and the expression of *Aldh1a2* and surface maturation markers including CD40, CD86, and MHC class II without reducing GM-CSF/Am80-induced CD103 expression (Fig. 1b,c). Similar results were obtained using pattern recognition receptor (PRR)-Ls including LPS, CpG ODN, and curdlan instead of Pam3CSK4 (see Supplementary Fig. S1). The PRR-L-treated DCs produced proinflammatory cytokines including IL-6, IL-12p40, IL-23p19, and TNF-α (Fig. 1d, also see Supplementary Fig. S1). However, it is known that CD103^+^ MLN-DCs from steady-state SPF mice weakly produce proinflammatory cytokines upon stimulation through CD40 or with LPS^9^. Therefore, alternative stimuli appeared to contribute to the development of CD103^+^ MLN-DCs with high ALDH activity (ALDH^high^) in naïve SPF mice.

To identify signals that enhance ALDH1A2 expression but induce low levels of proinflammatory cytokine production, we first hypothesized that CD103 might be involved in the induction of ALDH1A2 expression in the intestinal DCs. Most ALDH1A2^+^ MLN-DCs and LP-DCs express CD103, whereas intestinal epithelial cells (IECs) express E-cadherin, which can bind to CD103^25^. Furthermore, IECs are known to promote ALDH1A expression in DCs^26^,^27^. Simultaneous stimulation of BM-DCs with GM-CSF/Am80 and immobilized E-cadherin/Fc chimera protein induced ALDH activity, but this strategy could not enhance the CD103 expression in ALDH^+^ cells (Fig. 1a). Conversely, stimulation of BM-DC with GM-CSF/Am80 for 2 days followed by 1-day culture with E-cadherin markedly enhanced ALDH activity and the expression of CD40, CD86, MHC class II, and *Aldh1a2* without suppressing the GM-CSF/Am80-induced CD103 expression (Fig. 1b,c). Similar results were obtained using RA instead of Am80 (data not shown). This protocol also enhanced CCR7 expression, which is required for the migration of LP-DCs to MLNs (Fig. 1b)^28^. Both *Aldh1a2* expression and ALDH activity in MLN-DCs were much higher than those in LP-DCs in naïve SPF mice, and were diminished in MLN-DCs and LP-DCs in GM-CSF-deficient mice (see Supplementary Fig. S2), suggesting that the present protocol may mimic the GM-CSF-dependent maturation process of migratory RA-producing DCs in the intestine. Unlike PRR-L, E-cadherin did not trigger GM-CSF/Am80-treated BM-DCs to produce proinflammatory cytokines (Fig. 1d). E-cadherin partly suppressed the Pam3CSK4–induced production of IL-12p40 and IL-23p19 but not that of IL-6. However, after treatment with E-cadherin, Pam3CSK4 induced much lower levels of these three cytokines, suggesting that the E-cadherin-treated ALDH1A2^high^CD103^+^ DCs do not become proinflammatory as easily as immature DCs.

As the small intestine is rich in TGF-β^29^, we also examined the effect of TGF-β on BM-DCs. The addition of TGF-β together with GM-CSF and Am80 to BM-DC cultures enhanced their expression of CD103 but rather suppressed ALDH activity and the expression of CD86 and *Aldh1a2* after subsequent culture with or without E-cadherin or Pam3CSK4 (see Supplementary Fig. S3).

### Integrin β1 contributes to the effect of E-cadherin on *Aldh1a2* expression and ALDH activity in GM-CSF/Am80-treated BM-DCs

**Figure 2:**
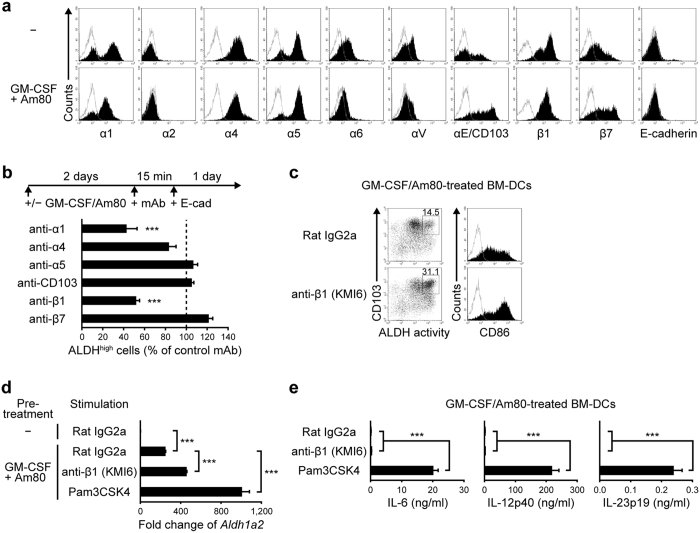
Integrin β1 contributes to the E-cadherin effect on GM-CSF/Am80-induced ALDH1A2 expression in DCs without inducing proinflammatory cytokines. (**a**) Representative flow cytometric profiles of integrin and E-cadherin expression on BM-DCs cultured for 2 days in the presence or absence of GM-CSF and Am80. Solid lines represent staining with isotype control mAbs. (**b**) Effects of blocking mAbs on the induction of ALDH^high^ DCs. GM-CSF/Am80-treated BM-DCs were pretreated for 15 min with blocking mAbs to integrins and stimulated for 1 day with immobilized E-cadherin/Fc. Percentages of ALDH^high^ cells were assessed by flow cytometry. Results are presented as the mean + SD (triplicate samples) relative to the control culture with the isotype control mAb. Statistical significance was determined using Student’s *t*-test. ***p < 0.001 versus control mAb. (**c**–**e**) GM-CSF/Am80-treated BM-DCs were stimulated for 1 day with immobilized anti-integrin β1 mAb (KMI6), or rat IgG2a isotype control mAb, or Pam3CSK4. (**c**) Representative flow cytometric profiles of ALDH activity and expression of indicated surface molecules. Solid lines represent staining with isotype control mAbs. (**d**) *Aldh1a2* expression assessed by real-time PCR. Relative expression levels are presented as the mean + SD of triplicate samples relative to that of the cells cultured in medium alone with rat IgG2a isotype control mAb. (**e**) Cytokine concentrations assessed by ELISA. Results are presented as the mean + SD of triplicate samples. Statistical significance was determined via the one-way ANOVA with Tukey–Kramer multiple comparisons test. ***p < 0.001. Data are representative of three independent experiments.

E-cadherin is known to interact with both CD103 and some other molecules including E-cadherin in a homophilic manner, integrin α2β1 (CD49b/CD29)^30^, and killer cell lectin-like receptor G1 (KLRG1)^31^,^32^. GM-CSF/Am80-treated BM-DCs expressed integrins α1 (CD49a), α4 (CD49d), α5 (CD49e), αE (CD103), β1, and β7 and weakly expressed integrins α6 (CD49f) and αV (CD51), whereas no expression of integrin α2, E-cadherin, and *Klrg1* was detected (Fig. 2a, and data not shown). Thus, it is unlikely that the homophilic interactions of E-cadherin or interactions with KLRG1 contributed to the effect of immobilized E-cadherin. Blocking mAbs to CD103 and integrin β7, which form a heterodimer, failed to suppress the effect of E-cadherin on ALDH activity (Fig. 2b). By contrast, blocking mAbs to integrins α1 (clone HMα1) and β1 (clone 9EG7) significantly suppressed this effect (Fig. 2b). The 9EG7 mAb binds to the active conformation of β1 integrins^33^. Another blocking mAb to β1 (clone HMβ1-1)^34^ also suppressed the effect, somewhat less effectively than 9EG7 (data not shown). However, when anti-integrin β1 mAbs (clones KMI6 and 9EG7) were immobilized on culture plates, they could enhance GM-CSF/Am80-induced ALDH activity and *Aldh1a2* expression without inducing proinflammatory cytokines similarly as immobilized E-cadherin (Fig. 2c–e, and data not shown). As integrins α1, α4, α5, α6, and αV can heterodimerize with integrin β1^35^, the heterodimer α1β1 appeared to contribute to the effect of E-cadherin.

### Conventional DCs from the LP, PPs, and MLNs and pre-DCs from spleens of normal mice express integrin β1, but only weakly express integrin α1

**Figure 3:**
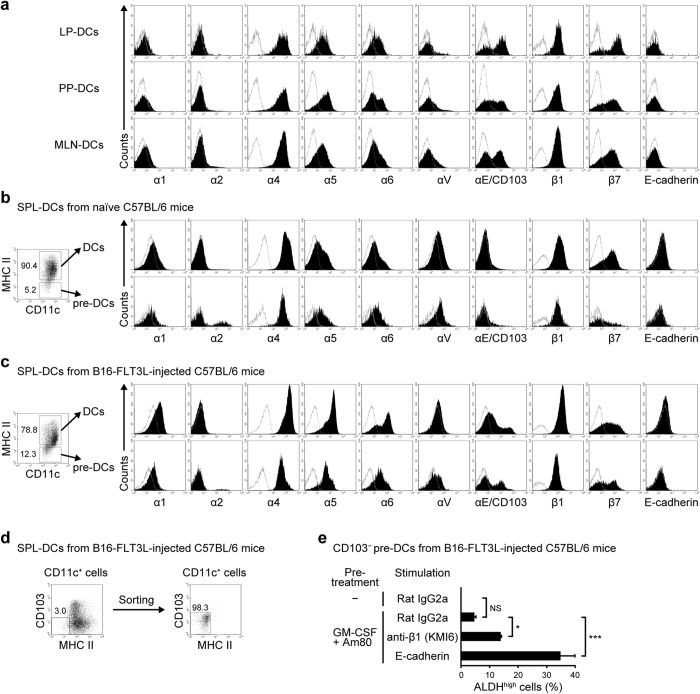
Integrin β1-mediated stimulation enhances ALDH activity in GM-CSF/Am80-treated pre-DCs in an integrin α1-independent fashion. (**a**) Representative flow cytometric profiles of integrin and E-cadherin expression on DCs from the small intestinal LP, PPs, and MLNs of C57BL/6 mice. (**b**–**c**) Representative flow cytometric profiles of integrin and E-cadherin expression on DCs (CD11c^+^MHC class II^+^) and pre-DCs (CD11c^+^ MHC class II^−^) from SPLs of naïve (**b**) or B16-FLT3L-injected (**c**) C57BL/6 mice. (**d**) Scheme illustrating the sorting of CD11c^+^MHC class II^−^CD103^−^ pre-DCs from SPLs of B16-FLT3L-injected C57BL/6 mice. (**e**) CD11c^+^MHC class II^−^CD103^−^ pre-DCs isolated from the SPL of B16-FLT3L-injected mice were cultured for 2 days with GM-CSF and Am80 and subsequently stimulated for 1 day with immobilized anti-integrin β1 mAb (KMI6), rat IgG2a isotype control mAb, or E-cadherin/Fc. Percentages of ALDH^high^ cells were assessed by flow cytometry. Results are presented as the mean + SD of triplicate samples. Statistical significance was determined via the one-way ANOVA with Tukey–Kramer multiple comparisons test. *p < 0.05, ***p < 0.001. Data are representative of three independent experiments.

Unlike FLT3-L–generated BM-DCs, conventional DCs in the LP, PPs, and MLNs of naïve mice only weakly expressed α1 (Fig. 3a). They expressed significant levels of integrins β1 and α4, and low levels of integrins α5, α6, and αV (Fig. 3a). However, no significant expression of E-cadherin and *Klrg1* was detected (Fig. 3a, and data not shown). Furthermore, integrin α1 expression on pre-DCs was also extremely weak (Fig. 3b,c). Therefore, integrin α1 appears to contribute little to the E-cadherin-dependent stimulation of intestinal DCs.

The analysis was performed using pre-DCs from the SPLs of normal mice and those of mice injected with B16-FLT3L cells. The B16-FLT3L injection did not affect integrin α1 expression on pre-DCs and DCs in the SPL, although it enhanced the expression of some integrins, including CD103. Stimulation of CD11c^+^MHC class II^−^CD103^−^ pre-DCs from the SPL of B16-FLT3L-injected mice (Fig. 3d) with GM-CSF/Am80 followed by 1-day culture in E-cadherin or anti-β1 mAb (KMI6)–coated plates enhanced the ALDH activity of the cells (Fig. 3e). The results suggest that integrin α1 expression is not essential for the effect of E-cadherin and the anti-β1 mAb.

### Integrin β1-mediated signaling enhances maturation and ALDH1A2 expression in BM-DCs, irrespective of the partner α subunit

**Figure 4:**
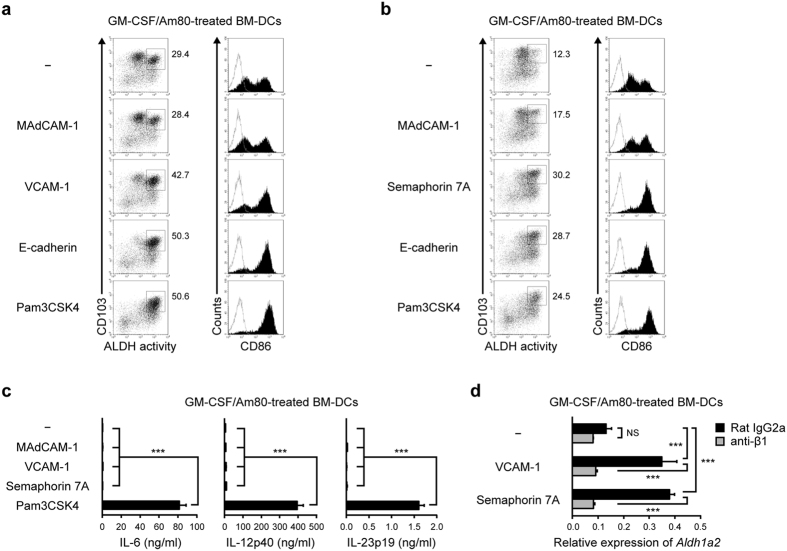
VCAM-1 and semaphorin 7 A enhance GM-CSF/Am80-induced ALDH1A2 expression in BM-DCs via integrin β1 without inducing proinflammatory cytokines. BM-DCs were cultured for 2 days with GM-CSF and Am80 and subsequently stimulated for 1 day with Pam3CSK4 or immobilized MAdCAM-1/Fc, VCAM-1/Fc, semaphorin 7 A/Fc, or E-cadherin/Fc. (**a**–**b**) Representative flow cytometric profiles of ALDH activity and CD86 expression are shown. (**c**) Cytokine concentrations in the culture supernatants were assessed by ELISA. (**d**) The blocking anti-integrin β1 (9EG7) mAb or control rat IgG2a mAb was added in the last 1-day culture. The expression of *Aldh1a2* was assessed by real-time PCR, and its level was normalized with *Rplp0* and quantified with the 2^−Δ*Ct*^ value. Results are presented as the mean + SD of triplicate samples. Statistical significance was determined by the one-way ANOVA with Tukey–Kramer multiple comparisons test. NS: not significant, ***p < 0.001.

An anti-integrin α4 mAb slightly suppressed the E-cadherin-dependent enhancement of ALDH activity in BM-DCs, albeit not significantly (Fig. 2b). On the other hand, immobilized MAdCAM-1/Fc chimera protein, a ligand for α4β7 but a poor ligand for α4β1, slightly enhanced ALDH activity and CD86 expression in GM-CSF/Am80-treated BM-DCs (Fig. 4a,b). As integrin β1 appeared to play an important role, we examined the effect of VCAM-1/Fc, which binds to integrin α4β1 (very late antigen-4, VLA-4) and α4β7^36^, and semaphorin 7 A/Fc, which binds to integrin β1 (heterodimerized with α1 or αV) and plexin C1^37^,^38^. Immobilized VCAM-1/Fc and semaphorin 7 A/Fc could significantly enhance ALDH activity and the expression of CD86 and *Aldh1a2* in GM-CSF/Am80-treated BM-DCs without inducing proinflammatory cytokines (Fig. 4). The enhanced *Aldh1a2* expression was significantly suppressed by the blocking anti-integrin β1 (9EG7) mAb (Fig. 4d). These results indicate that integrin β1-mediated signaling commonly contributes to the effects of E-cadherin, VCAM-1, and semaphorin 7 A, irrespective of the partner α subunit.

### FAK and its downstream signaling molecules are involved in the E-cadherin- and integrin β1-mediated generation of mature ALDH1A2^high^ DCs

**Figure 5:**
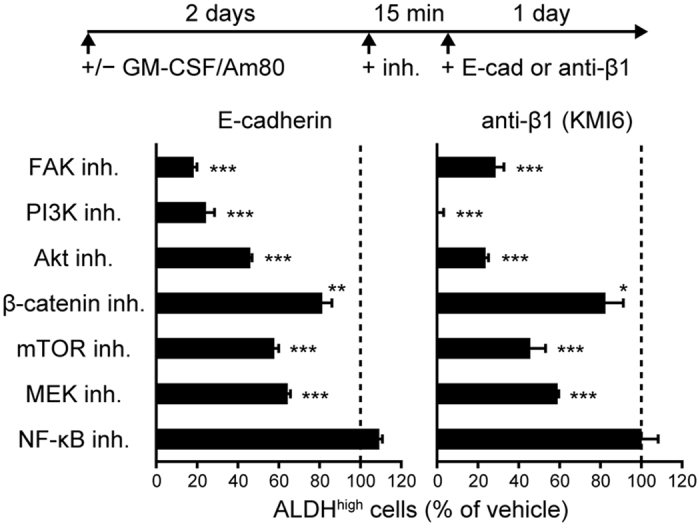
FAK and its downstream signaling molecules contribute to the E-cadherin– and integrin β1–mediated generation of ALDH1A2^high^ DCs. GM-CSF/Am80-treated BM-DCs were pretreated for 15 min with inhibitors of FAK, PI3K, Akt, β-catenin, mTOR, MEK/ERK, and NF-κB and stimulated for 1 day with immobilized E-cadherin/Fc or anti-integrin β1 (KMI6). Percentages of ALDH^high^ cells were assessed by flow cytometry. Results are presented as the mean + SD (triplicate samples) relative to the control culture treated with the vehicle and are representative of three independent experiments. Statistical significance was determined using the Student’s *t*-test. *p < 0.05, **p < 0.01, ***p < 0.001 versus vehicle.

Integrin-dependent signaling often involves the activation of FAK, also known as protein tyrosine kinase 2, and the subsequent activation of PI3K and Akt, also known as protein kinase B^39^. The FAK inhibitor PF 573228, the PI3K inhibitor LY294002, and the Akt inhibitor triciribine significantly prevented immobilized E-cadherin or anti-integrin β1 mAb (KMI6) from enhancing ALDH activity in GM-CSF/Am80-treated BM-DCs (Fig. 5). The MEK inhibitor PD98059 and the mTOR inhibitor rapamycin moderately suppressed the enhancement. The β-catenin inhibitor ICG-001 weakly suppressed this enhancement, and an NF-κB inhibitor failed to suppress it. These results support that the E-cadherin-dependent effect on DCs involves integrin-dependent signaling.

### ALDH1A2^high^ DCs generated by GM-CSF/Am80 and E-cadherin induce Foxp3^+^ T cells, but not Th1 or Th17 cells, and alleviate colitis

**Figure 6:**
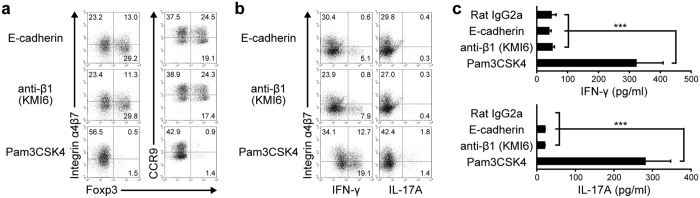
ALDH1A2^high^ DCs generated with GM-CSF/Am80 and E-cadherin induce gut-tropic Foxp3^+^ T cells but not Th1 or Th17 cells. GM-CSF/Am80-treated BM-DCs were stimulated for 1 day with immobilized E-cadherin/Fc, immobilized anti-integrin β1 mAb (KMI6), or Pam3CSK4, treated for 2 h with the OVA peptide P323-339, washed, and then cocultured for 5 days with naïve CD4^+^ T cells from OT-II/Rag2^−/−^ mice in the presence (**a**) or absence (**b**–**c**) of TGF-β. (**a**) Representative flow cytometric profiles of intracellular Foxp3 expression and surface expression of integrin α4β7 or CCR9 of gated CD4^+^ T cells. (**b**) Representative flow cytometric profiles of intracellular expression of IFN-γ or IL-17A and surface expression of integrin α4β7 of CD4^+^ T cells restimulated for 5 h with PMA and ionomycin. (**c**) Cytokine concentrations in the supernatants of DC-T cell cocultures were assessed by ELISA. Results are presented as the mean + SD of triplicate samples. Statistical significance was determined using the one-way ANOVA with Tukey–Kramer multiple comparisons test. ***p < 0.001. Data are representative of three independent experiments.

When GM-CSF/Am80-treated BM-DCs were subsequently stimulated with the TLR2/1-L Pam3CSK4, they acquired the capacity to induce the differentiation of naïve CD4^+^ T cells to IFN-γ-producing T cells and IL-17A-producing T cells but not to Foxp3^+^ T cells even in the presence of TGF-β (Fig. 6). Significant populations of these IFN-γ^+^ T cells and IL-17A^+^ T cells expressed integrin α4β7 (Fig. 6b). Similar results were obtained by using other PRR-Ls including LPS, CpG ODN, and curdlan (data not shown). On the contrary, when GM-CSF/Am80-treated BM-DCs were subsequently stimulated with E-cadherin or the anti-integrin β1 mAb (KMI6), these DCs could efficiently induce Foxp3^+^ T cells in the presence of TGF-β, but they induced few to no IFN-γ− or IL-17A–producing T cells (Fig. 6). Therefore, E-cadherin- or integrin β1-dependent regulation of proinflammatory cytokine production appears to be critical for inducing regulatory T cells and suppressing proinflammatory T cell differentiation. A significant population of the induced Foxp3^+^ T cells expressed integrin α4β7 and CCR9 (Fig. 6a), suggesting that gut-tropic iTregs were induced.

**Figure 7:**
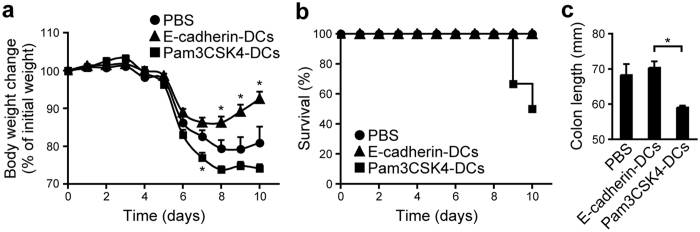
ALDH1A2^high^ DCs generated by treatment with GM-CSF/Am80 and E-cadherin alleviate DSS-induced acute colitis. Male C57BL/6 mice aged 8–9 wk were administered 2% DSS in their drinking water ad libitum for 4 days, followed by feeding with regular drinking water. On days 0 and 2, the mice were injected intraperitoneally with PBS alone or BM-DCs cultured for 2 days in the presence of GM-CSF and Am80 and subsequently stimulated for 1 day with immobilized E-cadherin/Fc or Pam3CSK4. Body weight changes and the survival ratios of individual groups of mice (n = 6–9) after DSS treatment are shown in (**a**) and (**b**), respectively. (**c**) Colon length was determined at day 10 after DSS treatment. Results are presented as the mean + SEM. Statistical significance was determined using the one-way ANOVA with Tukey–Kramer multiple comparisons test. *p < 0.05 (versus PBS in (**a**)).

To examine whether the E-cadherin–induced ALDH1A2^high^CD103^+^ DCs ameliorate inflammatory bowel diseases, we adoptively transferred them into DSS–induced colitis model mice. These DCs significantly suppressed DSS–induced body weight loss, without completely preventing it (Fig. 7a). Contrarily, DCs treated with Pam3CSK4 enhanced body weight loss, mouse mortality, and colon shortening (Fig. 7), suggesting that the maturation signal for GM-CSF– and RA–induced semi-mature DCs is critical for determining their proinflammatory or anti-inflammatory nature. The results collectively indicate that the sequential stimulation of FLT3-L-generated BM-DCs with GM-CSF/Am80 and E-cadherin efficiently induce anti-inflammatory DCs that can induce gut-tropic regulatory T cells.

## Discussion

The combination of GM-CSF and RA or Am80 cooperatively enhanced ALDH1A2 expression in pre-DCs as well as BM-DCs, and induced CD103 expression after 2 days of culture. Both GM-CSF and RA appear to be constantly available in the small intestine and MLNs. However, the expression levels of both ALDH1A2 and CD86 in GM-CSF/RA- or GM-CSF/Am80-treated BM-DCs were much lower than those in ALDH1A2^high^ MLN-DCs. Therefore, to mimic the generation of ALDH1A2^high^ MLN-DCs, additional signals are likely to be required for enhancing their ALDH1A2 expression and maturation. We previously employed TLR-Ls with GM-CSF for promoting the maturation and ALDH1A2 expression in BM-DCs^14^. Indeed, TLR-L or dectin-L enhanced their maturation and ALDH1A2 expression after 2 days of stimulation with GM-CSF and Am80 as well. However, they induced proinflammatory cytokine production, and the treated DCs could not induce Foxp3^+^ T cells, but induced IFN-γ^+^ T cells and a small number of IL-17A^+^ T cells bearing the gut-homing receptor integrin α4β7. Although RA can suppress the differentiation of both Th1 and Th17 cells^40^, certain levels of RA appear to be essential for Th1 cell lineage stability^8^, for Th17 differentiation^41^, and for the induction of gut-homing Th17^42^.

Contact between DCs and IECs that express E-cadherin is known to enhance ALDH1A2 expression in DCs^26^,^27^. Accordingly, our preliminary results indicated that co-culture of GM-CSF/Am80-treated BM-DCs with the IEC line CMT93 enhanced the induction of ALDH^high^ DCs, and that a blocking Ab to integrin β1 inhibited the effect. Furthermore, we found that stimulation of GM-CSF/Am80-treated BM-DCs with immobilized E-cadherin significantly enhanced their maturation and ALDH1A2 expression as well as their ability to induce Foxp3^+^ T cell differentiation in the presence of TGF-β. The results led us to speculate that semi-mature LP-DCs may become anti-inflammatory mature DCs by interacting with E-cadherin on IECs in steady state but become proinflammatory mature DCs by interacting with pathogen–derived PRR-L.

No evidence was obtained for the role of CD103 in the effect of E-cadherin. On the contrary, a blocking Ab to integrin β1 inhibited the effect, and immobilized anti-integrin β1 Ab could mimic the effect, suggesting that E-cadherin stimulated DCs partly through cross-linking of integrin β1. Accordingly, immobilized VCAM-1/Fc and semaphorin 7 A/Fc, which binds to integrin β1, could induce similar effects, and a blocking Ab to integrin β1 suppressed the effects. Semaphorin 7 A is expressed on IECs and activated T cells^37^,^38^, whereas VCAM-1 is mainly expressed on endothelial cells in the intestine^43^. Therefore, semaphorin 7 A but not VCAM-1 may contribute to the DC maturation step. Heterodimers of integrin β1 and some α integrins can bind to extracellular matrix glycoproteins including collagens, fibronectins, and laminins^36^. Extracellular matrix molecules are abundant in the intestinal LP and thus may partly contribute to steady-state DC maturation.

In addition to E-cadherin, TLR-Ls, and dectin-Ls, other stimuli may also contribute to the maturation or maintenance of ALDH1A2^+^ DCs. It has been revealed that 4-1BB (CD137) expressed on ALDH^high^CD103^+^ MLN-DCs plays a role in the maintenance of their ALDH1A2 expression^44^. In our culture system, preliminary results indicated that 4-1BB expression was induced in a subset of BM-DCs after culturing with GM-CSF for 2 days, but that its expression was significantly suppressed by adding Am80. It remains to be clarified whether 4-1BB-mediated signaling and integrin β1-mediated signaling interact with each other in the regulation of ALDH1A2 expression and DC maturation. It was recently reported that culturing BM cells with the combination of FLT3-L and GM-CSF for 16 days efficiently induced CD11c^high^B220^−^CD103^high^ conventional DCs^45^. Although these DCs did not express maximal levels of ALDH1A2, they appeared to induce T cell-mediated protective immunity *in vivo*. Resident DCs may also intrinsically reduce the severity of DSS colitis^46^. However, it has been reported that DSS induces production of proinflammatory cytokines by BM-DCs generated with GM-CSF and IL-4^47^. Furthermore, intestinal inflammation may abrogate the tolerogenic properties of CD103^+^ MLN-DCs, including *Aldh1a2* expression^48^. However, we found that inoculation of mature ALDH1A2^high^ DCs stimulated by E-cadherin *in vitro* could significantly suppress DSS–induced weight loss but that mature ALDH1A2^high^ DCs stimulated with Pam3CSK4 significantly enhanced body weight loss, colon shortening, and mortality. Although mature DCs have diminished capacity for macropinocytosis, they can continue to capture and present Ag *in vivo*^49^,^50^. The maturation levels of DCs for inoculation might be important for the maintenance of their anti-inflammatory capacity *in vivo*. GM-CSF/Am80-treated semi-mature BM-DCs could easily become proinflammatory or anti-inflammatory depending on the subsequent stimulation. After stimulation with E-cadherin, these DCs appeared to become more resistant to TLR stimulation to trigger proinflammatory cytokine production. In addition, these DCs appeared to be capable of inducing iTregs that expressed the gut-homing receptors integrin α4β7 and CCR9. DSS treatment is known to enhance the expression of the α4β7 ligand MAdCAM-1 in the colon^51^. Although the CCR9 ligand CCL25 is not significantly expressed in the healthy colon unlike MAdCAM-1, DSS treatment induces CCL25 expression in the inflamed colon, and CCL25/CCR9 interactions regulate inflammatory immune responses in the large intestinal mucosa^52^. As monocytes/macrophages and neutrophils are major colitogenic effector cells in DSS-treated mice^53^, the effect of the *in vitro*–generated anti-inflammatory DCs might be indirect through induction of gut-homing Tregs. In addition, RA produced by the inoculated DCs might contribute to the attenuation of intestinal inflammation by promoting IL-22 synthesis^54^.

Collectively, the present study suggests that FLT3-L-generated immature BM-DC acquire CD103 expression and moderate levels of RA-producing capacity upon stimulation with GM-CSF/RA or GM-CSF/Am80, and that subsequent stimulation with immobilized E-cadherin efficiently generates mature RA-producing anti-inflammatory DCs. It is likely that integrin β1-mediated signaling contributes to the E-cadherin effect and similar effects of other integrin β1-binding molecules. However, further studies are required to clarify the role of integrin β1-mediated signaling in the *in vivo* generation of intestinal ALDH^high^CD103^+^ DC.

## Additional Information

**How to cite this article**: Yokota-Nakatsuma, A. *et al*. Beta 1-integrin ligation and TLR ligation enhance GM-CSF–induced ALDH1A2 expression in dendritic cells, but differentially regulate their anti-inflammatory properties. *Sci. Rep.* **6**, 37914; doi: 10.1038/srep37914 (2016).

**Publisher's note:** Springer Nature remains neutral with regard to jurisdictional claims in published maps and institutional affiliations.

## Supplementary Material

Supplementary Information
