# Supplementary material for: Beta 1-integrin ligation and TLR ligation enhance GM-CSF–induced ALDH1A2 expression in dendritic cells, but differentially regulate their anti-inflammatory properties
Source: Sci Rep. 2016 Nov 29;6:37914. doi: 10.1038/srep37914 (PMC5126582; doi:10.1038/srep37914)
Supplement: Supplementary Information [file srep37914-s1.pdf]

Beta 1-integrin ligation and TLR ligation enhance GM-CSF–induced ALDH1A2 expression in dendritic cells, but differentially regulate their anti-inflammatory properties

Aya Yokota-Nakatsuma[1,2], Yoshiharu Ohoka[1,2], Hajime Takeuchi[1,2], Si-Young Song[2,3], Makoto Iwata[1,2*]

[1]Laboratory of Immunology, Kagawa School of Pharmaceutical Sciences, Tokushima Bunri University, Shido, Sanuki-shi, Kagawa, Japan

[2]Japan Science and Technology Agency, CREST, Chiyoda-ku, Tokyo, Japan

[3]Institute of Neuroscience, Tokushima Bunri University, Shido, Sanuki-shi, Kagawa, Japan.

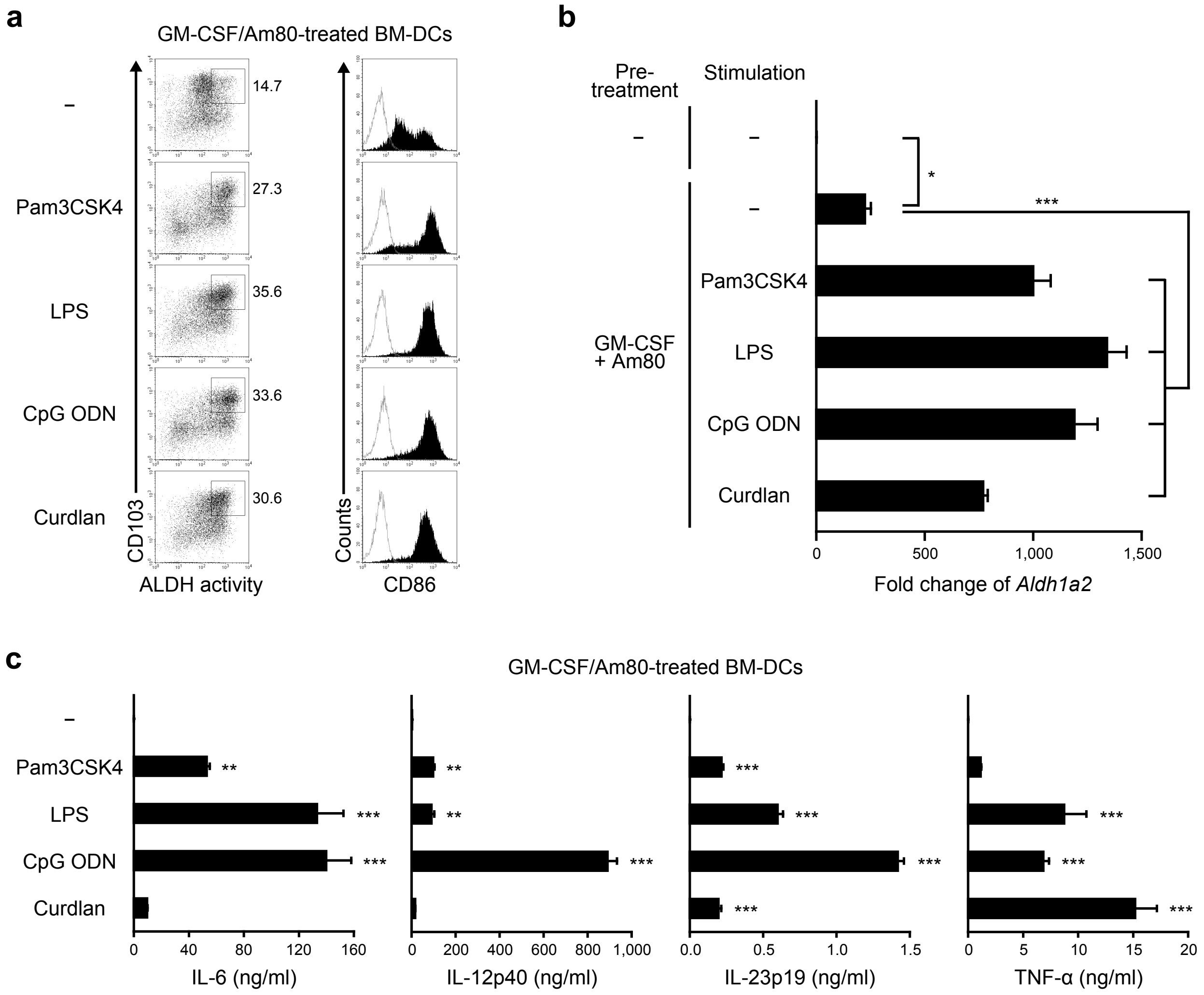

Supplementary Figure S1 : Delayed stimulation of BM-DCs with PRR-L 2 days after the start of culture with GM-CSF and Am80 markedly enhances ALDH1A2 expression without reducing CD103 expression. BM-DCs were cultured for 2 days with GM-CSF and Am80 and subsequently stimulated for 1 day with Pam3CSK4, LPS, CpG ODN, or curdlan. (**a**) Representative flow cytometric profiles of ALDH activity and expression of indicated surface molecules are shown. (**b**) *Aldh1a2* expression was assessed by real-time PCR. Relative expression levels are presented as the mean + SD of triplicate samples relative to that of the cells cultured in medium alone. (**c**) Cytokine concentrations in the culture supernatants were assessed by ELISA. Results are presented as the mean + SD of triplicate samples. Statistical significance was determined by the one-way ANOVA with Tukey–Kramer multiple comparisons test. *$p < 0.05$, **$p < 0.01$, ***$p < 0.001$ (versus control in (**c**)).

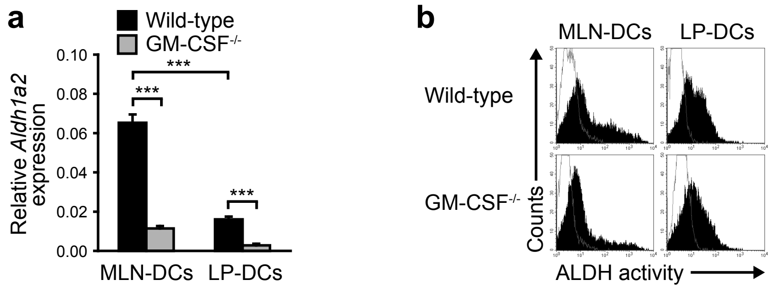

Supplementary Figure S2 : GM-CSF plays pivotal roles in the ALDH1A2 expression in MLN-DCs and LP-DCs. (**a**) *Aldh1a2* expression in DCs from MLNs and the small intestinal LP of wild-type C57BL/6 mice and GM-CSF$^{-/-}$ mice. Relative expression levels are quantified with the $2^{-\Delta Ct}$ value and presented as the mean + SD of triplicate samples. Statistical significance was determined using the one-way ANOVA with Tukey–Kramer multiple comparisons test. ***$p < 0.001$. (**b**) Representative flow cytometric profiles of ALDH activity in cells incubated with ALDEFLUOR in the presence (solid lines) or absence (shaded histograms) of the ALDH inhibitor diethylaminobenzaldehyde. Data are representative of three independent experiments.

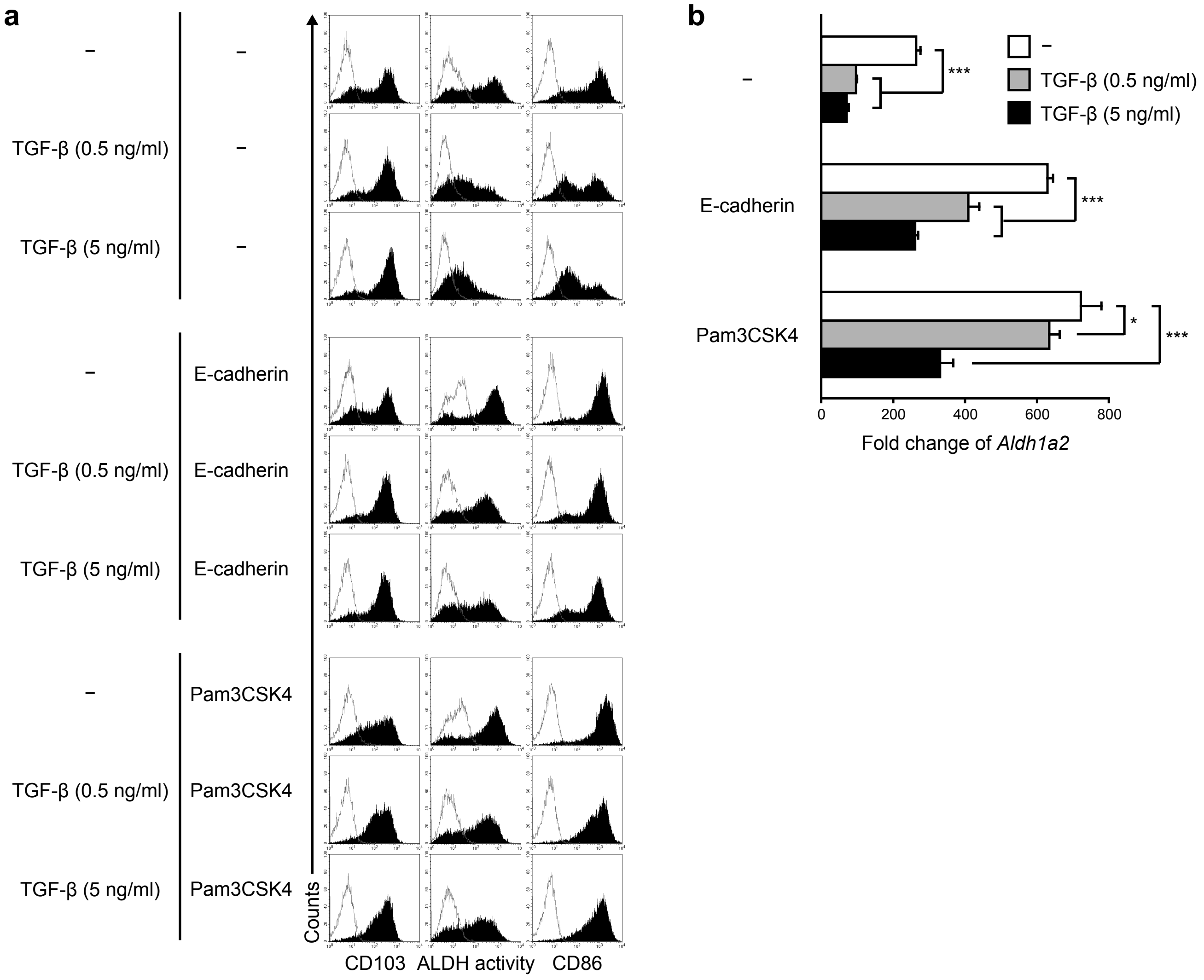

Supplementary Figure S3 : Addition of TGF-β together with GM-CSF and Am80 to BM-DC culture enhances CD103 expression, but suppresses ALDH activity and the expression of *Aldh1a2* expression and the surface maturation marker CD86. BM-DCs were cultured for 2 days in the presence of GM-CSF and Am80 with or without TGF-β and subsequently stimulated for 1 day with E-cadherin or Pam3CSK4. (**a**) Representative flow cytometric profiles of ALDH activity and expression of indicated surface molecules are shown. (**b**) *Aldh1a2* expression was assessed by real-time PCR. Relative expression levels are presented as the mean + SD of triplicate samples relative to that of the cells cultured in medium alone. Statistical significance was determined by the one-way ANOVA with Tukey–Kramer multiple comparisons test. $^*p < 0.05$, $^{***}p < 0.001$.
